# Identification of mutations in the HVR1 and PKR-BD regions in HCV-infected patients resistant to PEG-IFNα/RBV therapy

**DOI:** 10.1007/s13353-014-0267-0

**Published:** 2015-01-15

**Authors:** M. Holysz, K. Bialas, P. Migdalski, D. Kmieciak, W. H. Trzeciak

**Affiliations:** 1 Department of Biochemistry and Molecular Biology, University of Medical Sciences, 6 Święcickiego St., 60-781 Poznań, Poland; 2 Department of Infectious Disease, University of Medical Sciences, 6 Święcickiego St., 60-781 Poznań, Poland

**Keywords:** HCV-1b quasi-species, High resolution melting, Ineffective PEG-IFNα/RBV therapy

## Abstract

The identification of mutations in the HVR1 region of hepatitis type C virus (HCV) is time-consuming and expensive, and there is a need for a rapid, inexpensive method of screening for these mutations to predict the ineffectiveness of pegylated interferon alpha combined with ribavirin (PEG-IFNα/RBV) therapy. The project was designed to evaluate the usefulness of the high resolution melting (HRM) technique to screen for mutation in the cDNAs encoding the HVR1 and protein kinase R-binding domain (PKR-BD) regions in a group of 36 patients infected with HCV and resistant to 12 months of combined therapy with PEG-IFNα/RBV. Viral RNA was isolated, reverse transcribed, and the fragments encoding the HVR1 and PKR-BD regions were polymerase chain reaction (PCR)-amplified, cloned, sequenced, and the melting profiles and the melting temperature (T_m_) were determined by the HRM technique. After the treatment, the melting profiles of HVR1 cDNAs revealed a dominant peak corresponding to the T_m_ of about 85 °C (HCVs85) in almost all patients. One or more minor peaks were also observed, indicating the existence of cDNA(s) of different T_m_. The HMR analysis suggested four typical forms of response to treatment. These suppositions were supported by sequencing. The HRM analysis revealed no changes in the melting profiles of PKR-BD cDNAs in the same patient before and after the therapy, suggesting that, within 12 months of treatment, new mutations were not introduced in PKR-BD. These findings were substantiated by sequencing. The HRM technique can be applied for the rapid screening for mutations in the cDNAs encoding the HVR and PKR-BD regions of HCV. We suggest that the detection of HCVs85 peak before the IFNα/RBV therapy might predict the ineffectiveness of treatment.

## Introduction

Infection with hepatitis type C virus (HCV) is an important epidemiologic problem and one of the most common causes of liver diseases worldwide. According to the World Health Organization (WHO), about 3 % of the world’s population has been infected with this virus and nearly 80 % of the infected individuals develop chronic liver disease, leading to liver cirrhosis and, in some cases, to hepatocellular carcinoma (WHO 2014).

Standard therapy of the disease, based on pegylated interferon alpha combined with ribavirin (PEG-IFNα/RBV), enables to achieve a sustained virologic response (SVR) only in at most 56 % of patients qualified for this type of treatment (Fried et al. 2002). The serine protease inhibitors telaprevir (TVR) or boceprevir (BEC) are presently added to standard PEG-IFNα/RBV treatment, resulting in improvement of SVR to over 70 % (Kozielewicz et al. 2012).

Numerous genetic studies have demonstrated that the effect of the therapy mostly depends on the genotype of the virus, as well as on its ability to create new strains (quasi-species) in the course of infection (Holland et al. 1992).

In Poland, about 90 % of patients are infected by the particularly dangerous HCV-1b subspecies (Brojer et al. 1996). In case of infection with this virus, only 46 % of patients can be completely cured (Brojer et al. 1996), while in patients infected with the virus of other genotypes, PEG-IFNα/RBV therapy is effective in 77 % of cases (Fried et al. 2002; Manns et al. 2001).

An important role in the response to therapy is played by envelope glycoproteins E1 and E2, responsible for the delivery of viral RNA to the target cells. These proteins contain hypervariable regions HVR1 and HVR2, respectively, which undergo extensive mutations during the course of infection, resulting in the generation of quasi-species, which can be eliminated during therapy and replaced by better evolutionary adjusted forms of the virus. This is considered a way of avoiding by the virus the host-derived immune pressure. As a result, the virus not only goes through the acute phase of the disease but also establishes itself in the chronic phase, and then persists, because specific antibodies directed against E2 cannot neutralize new epitopes created by changes emerging within the E2 structure.

The therapeutic effect of IFNα depends on the induction of several antiviral genes via the IFN signaling pathway. The binding of INF to specific receptor leads to the phosphorylation of STAT1 and STAT2 proteins by tyrosine kinases, Jak1 and Tyk2. This enables the formation of STAT1/STAT2 heterodimer, which binds to the p48 protein and forms a complex, called INF-stimulated gene factor 3 (Kmieciak 2005). This complex, after reaching the nucleus, binds with the INF-stimulated response element (ISRE) in order to induce transcription of several antiviral genes, including that coding for the double-stranded RNA-activated protein kinase (PKR), which inhibits protein synthesis by phosphorylation of the initiation factor 2α (eIF2α).

In spite of high expression of the PKR, the translation of viral proteins is not necessarily impaired, since the PKR shows significant affinity to the sequences of several viral proteins (Kmieciak 2005). One of these sequences, located in the C-terminal region of NS5A [last 66 amino acids (aa)] and called the protein kinase R-binding domain (PKR-BD), binds and inactivates PKR (He et al. 2001). Therefore, the occurrence of the mutations in the PKR-BD region, which affects the binding of PKR, might influence the synthesis of viral proteins and, thus, the effectiveness of PEG-IFNα/RBV therapy.

The methods recently used to identify quasi-species of the virus are based on reverse transcription followed by amplification of cDNA by real-time polymerase chain reaction (qPCR), cloning, and sequencing. We have recently proposed applying the high resolution melting (HRM) technique for rapid screening for mutations in the HVR1 and PKR-BD regions in one of our patients in order to evaluate the effectiveness of the PEG-IFNα/RBV therapy (Holysz et al. 2014).

In the present study, we have screened for mutations in the HVR1 and PKR-BD regions in a group of patients resistant to PEG-IFNα/RBV therapy by the HRM method and have chosen several patients for identifying mutations by cloning and sequencing to evaluate the compatibility of results obtained by the two methods.

## Materials and methods

### Patients and treatment

Thirty-six patients treated at the Department of Infectious Diseases of the University of Medical Sciences in Poznań were enrolled in the study. The patients underwent a 12-month combined therapy with 180 μg PEG-IFNα-2a (Hoffmann-La Roche, Nutley, NJ, USA), given once a week as a subcutaneous injection, plus a daily oral dose of RBV (Hoffmann-La Roche, Nutley, NJ, USA) 600–1,200 mg, according to the body weight.

The sera from each patient were analyzed before the treatment and after 12 months of ineffective therapy. The Ethical Committee of the University approved the study and written consent was obtain from each patient. The study adheres to the principles of the Declaration of Helsinki.

The level of inflammation (grading) and fibrosis (staging), indicating chronic hepatitis, were confirmed by histological examinations of the samples taken by liver biopsy. Hepatitis B virus (HBV) or other hepatotropic viruses infections, as well as chronic alcohol abuse, were excluded.

### Determination of viral load

Viral load was determined at the beginning and 12 months after the therapy by reverse transcription (RT), followed by quantitative PCR (Amplicor Monitor v.2.0; Roche Diagnostics, Branchburg, NJ, USA; linear range: 600–700 IU/ml). A qualitative RT-PCR assay (COBAS AmpliScreen v.2.0 HCV test; Roche Diagnostics; detection limit 100 IU/ml) was carried out after treatment.

Total RNA from the sera was prepared using a modified phenol–chloroform method (Chomczynski and Sacchi 1987) using TRIzol Reagent (Invitrogen Life Technologies, Carlsbad, CA, USA), as indicated by the manufacturer. The RT was performed in a 25-μl reaction mixture containing 10 μl RNA, 6 μl 5× buffer (Invitrogen Life Technologies, Carlsbad, CA, USA), 1 μl RNase inhibitor (RNAzin; Promega, Madison, WI, USA), 3 μl 0.1 M dithiothreitol, 1.5 μl 2.5 mM dNTPs (Sigma-Aldrich, St. Louis, MO, USA), 6.5 μl H_2_O, 1 μl (200 U) Moloney murine leukemia virus reverse transcriptase (Invitrogen Life Technologies), and 1 μl (50 pmol) of the HCV-specific primers (Table 1), designed according to the reference sequence D90208. The reaction mixture was incubated for 90 min at 37 °C, followed by 5 min of boiling and subsequent chilling on ice.

**Table 1:** List of primers used

Primer		Sequence 5′–>3′	Used for
ppo2R	R	GCCGAAACGGTCGGTCGT	Reverse transcription of HVR1 mRNA
ppo4R	R	TWGTRGCGAGCTCCGCCAAG	Reverse transcription of PKR-BD mRNA
HVR1	F	AGGTBYTGATTGTGATGCTAC	Amplification of HVR1 cDNA
	R	AGTCATTGCAGTTCAGGGC	
HVR1-C	F	AAGCTTAGGTBYTGATTGTGATGCTAC	Cloning of HVR1 cDNA (*HindIII* and *PstI* restriction sites are underlined)
	R	TGCTGCAGTCATTGCAGTTCAGGGC	
PKR-BD	F	TCCTTGGCCAGCTCHTCAGC	Amplification of PKR-BD cDNA
	R	TGGGCAWCGCTGGRGGGAA	
PKR-BD-C	F	ATCGATTCCTTGGCCAGCTCHTCAGC	Cloning of PKR-BD cDNA (*ClaI* and *XhoI* restriction sites are underlined)
	R	CTCTCGAGACMACCGTCCKCTTCYTCCG	

*F*, forward; *R*, reverse

### qPCR HRM

HRM analysis was employed to identify the HCV strains by using sets of the HVR1-specific primers (Table 1). The reaction mixture contained: 1 μl of cDNA, 5 μl of LightCycler® 480 High Resolution Melting Master Mix (2 × conc.) (Roche Diagnostics, Germany), primers dissolved to the final concentration of 0.5 μM, and MgCl_2_ at the final concentration of 3 mM. qPCR reactions and HRM analysis were performed on the LightCycler 480 II Instrument (Roche Diagnostics, Germany). The qPCR profile comprised one initial cycle of 95 °C for 10 min (Hot Start activation) and was followed by 40 cycles of 95 °C for 10 s, 60 °C for PKR-BD or 55 °C for HVR1 for 20 s, 72 °C for 10 s, and with a single fluorescence measurement. After the amplification step, HRM analysis was performed using a melting profile from temperature 70 to 99 °C rising at 0.02 °C per s (continuous fluorescent assay). The melting curves were analyzed with the use of the LightCycler Software Version 1.5 using Gene Scanning module (Roche Diagnostics, Germany). Corresponding annealing temperatures of the primers for both PKR-BD and HVR1 were determined based on qPCR amplification with annealing temperature gradient using a CFX96 instrument (Bio-Rad, USA).

### Cloning and sequencing

A semi-nested PCR was utilized to amplify the PKR-BD and HVR1 coding sequences. A 4-μl quantity of cDNA was added to 16 μl of PCR mixture containing: 1.6 μl 2.5 mM dNTPs (Sigma-Aldrich, St. Louis, MO, USA), 0.4 μl of each 20 μM primer (Table 1), 2 μl 10× buffer, and 0.2 μl 3 U/μl high-fidelity Pfu DNA Polymerase (Promega, USA).

Cycling parameters for amplification were as follows: 1 min at 94 °C, 40 cycles of 1 min at 94 °C, 2 min at 45 °C, 3 min at 72 °C, and a single final extension step for 7 min at 72 °C. The DNA products (517 nt) were purified, digested with relevant restriction enzymes: PKR-BD: *ClaI* and *XhoI*; HVR1: *HindIII* and *PstI* (Roche Diagnostics GmbH, Mannheim, Germany), ligated into a pBluescript SK+/− (Stratagene, La Jolla, CA, USA), digested as above, and used to transform *Escherichia coli* cells (XL1-Blue strain, Stratagene). The recombinant colonies were then amplified in 2 ml of the LB broth (Invitrogen Life Technologies, Carlsbad, CA, USA), followed by the isolation of plasmids. The recombinant plasmids were subjected to sequencing, and the nt sequence was converted to an aa sequence by using the OMIGA 2.0 program (Genetics Computer Group, Oxford Molecular Company, Madison, WI, USA, 1999).

## Results

### Screening for mutations in the HVR1 cDNA by HRM analysis

Mutations can be visualized as changes in the melting profiles of the specific cDNAs, recorded at the beginning and after 12 months of PEG-IFNα/RBV therapy. As a source of the virus, we used serum samples derived from 36 patients infected with HCV, before and after 12 months of unsuccessful therapy.

The HMR analysis, covering the HVR1 region, which encodes 27 N-terminal amino acids of E2, revealed a single, dominating peak with the melting temperature (T_m_) of 85 °C (HCVs85) in most samples. The HCVs85 peak was recorded in the majority of samples obtained after the PEG-IFNα/RBV therapy compared with samples before therapy. According to the character of changes observed in the melting profiles of cDNAs, several typical forms of responses to the therapy can be distinguished.

### Characteristic melting profiles of the cDNA encoding HVR1

**Fig. 1:**
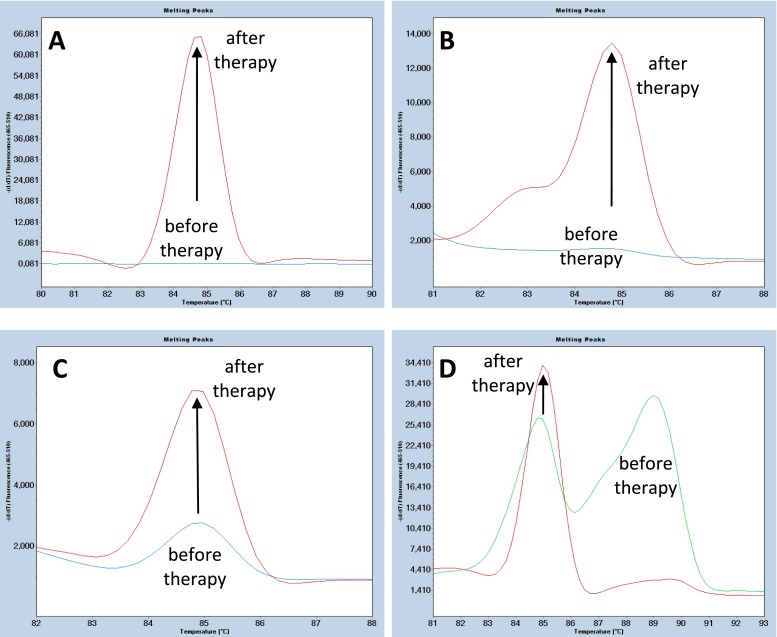
Typical melting peaks and profiles of the HVR1 amplification products before and after therapy: **a** appearance of the HCVs85 peak after therapy; **b** appearance of the HCVs85 peak and emergence of other peaks corresponding to other strains of the virus after therapy; **c** rise in the HCVs85 peak after therapy; **d** rise in the HCVs85 peak accompanied by appreciable reduction of peaks corresponding to initial strains after therapy

In about 50 % of the patients, the HCVs85 peak was undetectable before therapy, but it appeared after the therapy alone (Fig. 1a) or it was accompanied by other peaks of different T_m_, representing different quasi-species of the virus, as judged from the shape of the melting curve (Fig. 1b). In about one quarter of the patients, the HCVs85 peak was clearly visible before the treatment and it was much higher after treatment (Fig. 1c). In the remaining about one quarter of the patients before therapy, the melting profile revealed two or more separate peaks: the HCVs85 peak, and a broad, asymmetric peak with a T_m_ of 89 °C, which almost disappeared after therapy, while the HCV85 peak was increased (Fig. 1d).

### Analysis of cDNA sequences encoding HVR1

The results of screening for mutations in the HVR1 region by HRM were confirmed by sequencing of the HVR1 cDNA extracted from individual clones and the cDNA sequence was converted into an aa sequence of the HVR1 protein.

**Fig. 2:**
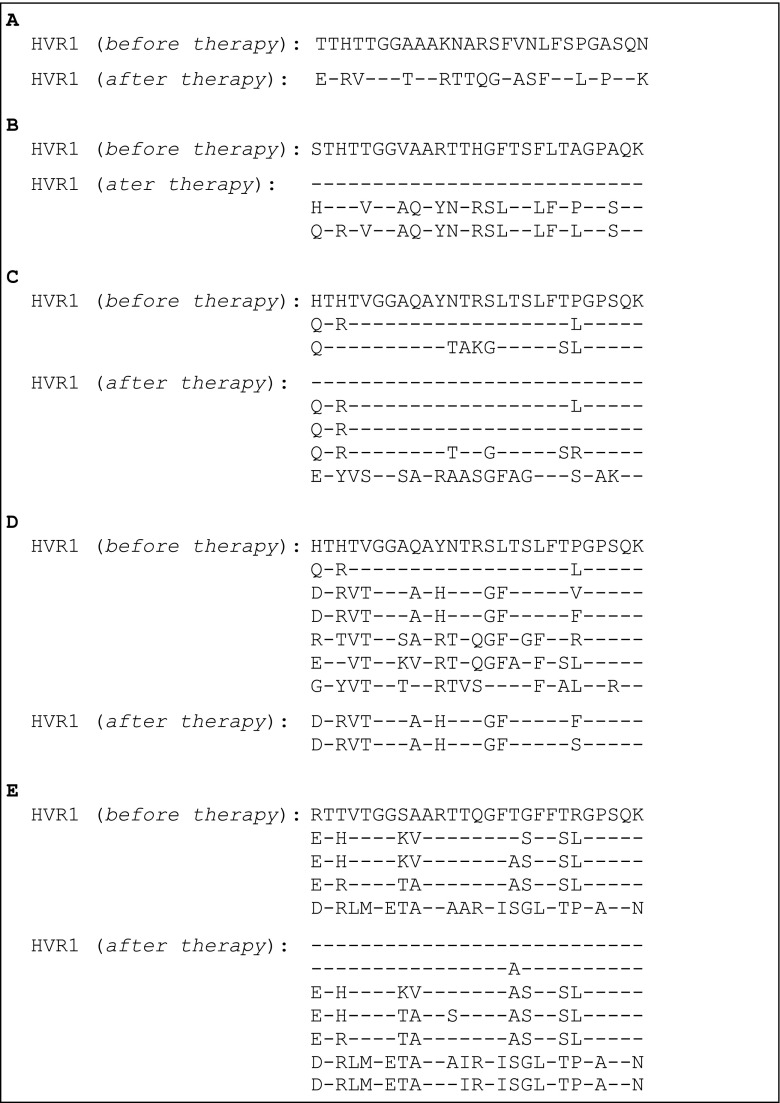
Comparison of amino acid sequences of the HVR1 region before and after therapy: **a** major changes in the HVR1 sequence after therapy; **b** emergence of additional strains of the virus after therapy; **c** appearance of additional strains before therapy and emergence of other strains of the virus after therapy; **d** reduction of initial strains after therapy; **e** change in the composition of strains after therapy

The aa sequence of the HVR1 region in one patient, predicted from the nucleotide sequence of the qPCR products cloned into the pSK Bluescript vector, showed a single species of the virus before the therapy, and only one species of very different sequence (the homology being lower than 40 %) after the therapy (Fig. 2a). The melting profile of HVR1 cDNA corresponding to sequencing analysis is presented in Fig. 1a.

In the other patient, the aa sequence after therapy revealed no changes in the sequence of the strain detected before therapy and the appearance of two accompanying strains of very different sequences from the initial strain (50 % homology) but 89 % homologous to each other (Fig. 2b). The corresponding melting profile of HVR1 cDNA is shown in Fig. 1b.

In yet another patient, the aa sequence of the HVR1 region before therapy revealed one major and two highly homologous minor strains of the virus. After therapy, the sequence of the major strain and one highly homologous strain remained unchanged, while two other strains highly homologous to the minor strains emerged. In addition, yet another strain, homologous to the HCV1a virus, was also detected (Fig. 2c). The results corresponding to the melting profile of HVR1 cDNA are shown in Fig. 1c.

In another patient, sequencing analysis of the HVR1 region revealed seven strains of the virus before therapy: two highly homologous (differing by three aa only), two differing by nine aa, but homologous to each other, and three differing from the major ones by 13, 14, and 14 aa, respectively. After therapy, one of the strains remained unchanged and another almost identical strain appeared. This indicated that, out of the seven strains, five were eliminated, meaning that they were sensitive to the therapy (Fig. 2d). The corresponding melting profile of HVR1 cDNA is shown in Fig. 1d.

In the next patient, sequencing analysis before therapy revealed one major and three highly homologous strains (differing by 7, 8, and 8 aa, respectively) plus yet another strain, which exhibited only 33 % homology to the major strain. After therapy, the major strain and all four homologous strains persisted, but an additional strain, differing only by 1 aa, was also present. Instead of an initial strain which exhibited low homology to the major one, after treatment, two strains almost identical to the original one and highly homologous to each other (over 96 % homology) were identified (Fig. 2e). This indicated that the initial strain of the virus was fully resistant to the therapy. The corresponding melting profile of HVR1 cDNA is shown in Fig. 1d.

### Screening for mutations in the PKR-BD cDNA by the HRM method

**Fig. 3:**
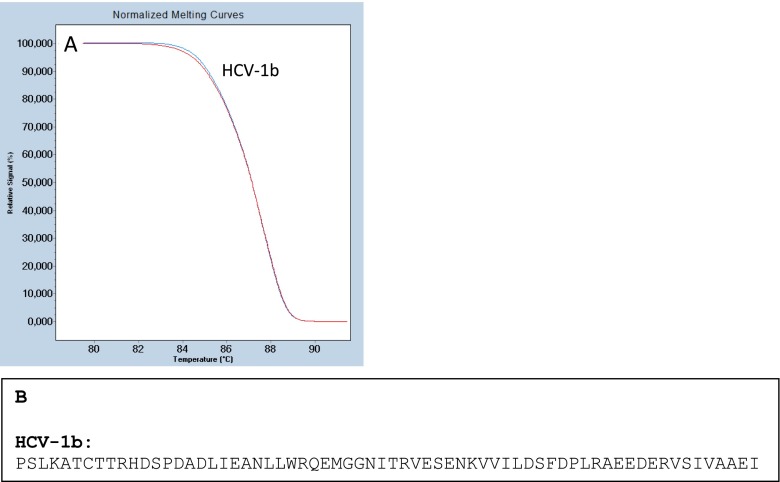
Melting profile of the PKR-BD amplification products before and after PEG-IFNα/RBV therapy: **a** typical melting profile for patients with HCV-1b subtype present before and after therapy; **b** the amino acid sequence of the C-terminal region of the NS5A protein, covering the PKR-BD region for HCV-1b

Screening for mutations in the cDNAs encoding PKR-BD revealed nearly identical melting profiles in all patients before the therapy, and the combined melting profiles after the therapy were identical to those recorded before the therapy (Fig. 3a). This strongly suggested that all the sequences of cDNAs were identical, or at least nearly identical, and no mutations to the fragment of the gene encoding PKR-BD were introduced during the therapy (Fig. 3b). Since the PKR-BD protein is highly conserved, we decided that cloning and sequencing of the PKR-BD cDNA will not provide valuable information as to the efficacy of treatment.

## Discussion

As a source of the virus, we used serum samples derived from 36 patients infected with HCV, before and after 12 months of unsuccessful therapy. As a screening method, we applied reverse transcription followed by amplification of cDNA by qPCR, connected with the analysis of melting profiles of the qPCR products, and measurements of the T_m_ by HRM. This technique is used mainly for the genotyping of single nucleotide polymorphisms (SNPs) and even a single nucleotide substitution can be detected. Therefore, we decided to use this method to screen for all sequence variations in the cDNAs spanning the HVR1 and PKR-BD regions of the HCV genome.

According to sequence diversity of different HCV-1b quasi-species, the analysis of melting profiles of HVR1 should show multiple amplification products identified as separate peaks of different T_m_. However, in almost all serum samples, we observed an amplification product which melts at about 85 °C.

In 17 out of 36 patients, the HCVs85 peak, undetectable before the therapy, appeared after unsuccessful treatment (alone or accompanied by other peaks), or at least the HCVs85 peak height was visibly increased after therapy (19 cases), while other peaks were eliminated or their height was decreased.

It has been recently reported in a group of 16 HIV and HCV co-infected subjects (Abdelrahman et al. 2015) infected with several variants of HCV1 that some patients were infected with two subtypes (1a and 1b) and only a single patient was infected with 1a and 4d. The investigators also found that minority strains (quasi-species), infrequent before therapy, reached up to 75–100 % of the total viral population after therapy. These results are strikingly similar to our observations made in 36 patients and strongly support our findings.

Another conclusion from both studies was that cloning greatly increases the sensitivity of the assay and enables the detection of quasi-species of the virus. Since in the above mentioned report (Abdelrahman et al. 2015) the Sanger sequencing technique alone did not allow the detection of quasi-species in all samples, they decided to use cloning and next-generation sequencing (NGS), which clearly showed quasi-species even before therapy and multiple strains after therapy. We have demonstrated that, due to cloning of the fragments made in our patients, even without the use of the NGS technique, we were able to show the presence of multiple quasi-species both before and after therapy. Considering the cost of NGS, application of this technique for clinical purposes is presently impractical. Cloning followed by Sanger sequencing or even analysis of the melting profiles of the fragments as a screening technique is sufficient to obtain enough information related to the therapy.

As evidenced from the sequencing data, the HCVs85 peak represents an average T_m_ of several strains of the virus. Domination of the HCVs85 peak might be explained by immunological pressure. The strains of T_m_ about 85 °C probably exhibit higher resistance to PEG-IFNα/RBV therapy than other quasi-species, which could have been eliminated. The observed changes in the aa sequence of HVR1 might reduce the recognition ability of the E2 antigens by anti-E2 antibodies and help the virus to escape from immunological pressure.

Analysis of the cDNA sequence of the PCR products covering the HVR1 region showed that the variants of HCV, which appear or sustain their presence in blood plasma despite the antiviral treatment, differ only in a single nucleotide, which results in, at most, a single aa substitution, while in most cases, the primary sequence remains unchanged. Therefore, the T_m_ of the HVR1 remained almost unchanged due to the character of nucleotide substitution. In most cases, we found that transversions within the complementary base pairs (e.g., AT↔TA or GC↔CG), usually affect the melting profile to a very small extent (less than 0.2 °C), which is impossible to recognize, even by HRM analysis. In our study, low heterogeneity of nucleotide sequences within HVR1 observed after therapy was associated with the gradual elimination of PEG-IFNα/RBV-sensitive variants, which were replaced by quasi-species that are more resistant to the therapy. This might have given rise to a therapy-resistant strain, melting at about 85 °C (HCVs85).

We also investigated the PKR-BD region (66 aa in length), comprising a 40 aa interferon sensitivity determining region (ISDR). Based on available data (Simmonds 2004), the sequence of PKR-BD is conservative. Besides HVR1, the regions that bind and inactivate PKR help to maintain the presence of HCV, despite the antiviral response of the immune system. HCV-1b is the only subtype that contains two regions which bind PKR: PePHD (in the E2 protein) and PKR-BD (in the NS5A protein). Berg et al. (2000) discovered that a higher frequency of mutations in the ISDR is associated with stronger response to therapy (Berg et al. 2000; Kmieciak et al. 2009). However, mutations in the additional 26 aa sequence, aside from the ISDR, were more frequent in patients resistant to therapy (Nousbaum et al. 2000; Enomoto et al. 1996). However, in all our patients, except for one case described elsewhere (Holysz et al. 2014), we have not found any changes in the aa sequence of PKR-BD. Similar results were obtained by other investigators (Aslan et al. 2004; Kmieciak et al. 2006).

The novel aspect of our study is the application of the HRM technique for the rapid screening for sequence variations in the HVR1 and PKR-BD regions of HCV. This technique has never been used for that purpose. The HMR method, although it applies for the analysis of any sequence changes and is rapid and inexpensive, has a serious disadvantage, because nonspecific fluorescent dyes are used, and, therefore, its application is limited. However, in spite of this disadvantage, after precise validation, it might be used in clinical virology as a screening method to assess the effectiveness of antiviral therapy. Our results suggest that the detection of HCVs85 in patients’ cDNA encoding HVR1 before therapy is initiated might predict the ineffectiveness of PEG-IFNα/RBV therapy alone.
